# Supplementary material for: OsLEA1b Modulates Starch Biosynthesis at High Temperatures in Rice
Source: Plants (Basel). 2023 Dec 4;12(23):4070. doi: 10.3390/plants12234070 (PMC10708107; doi:10.3390/plants12234070)
Supplement: Supplementary file 1 [file plants-12-04070-s001.zip › plants-2739884-supplementary.pdf]

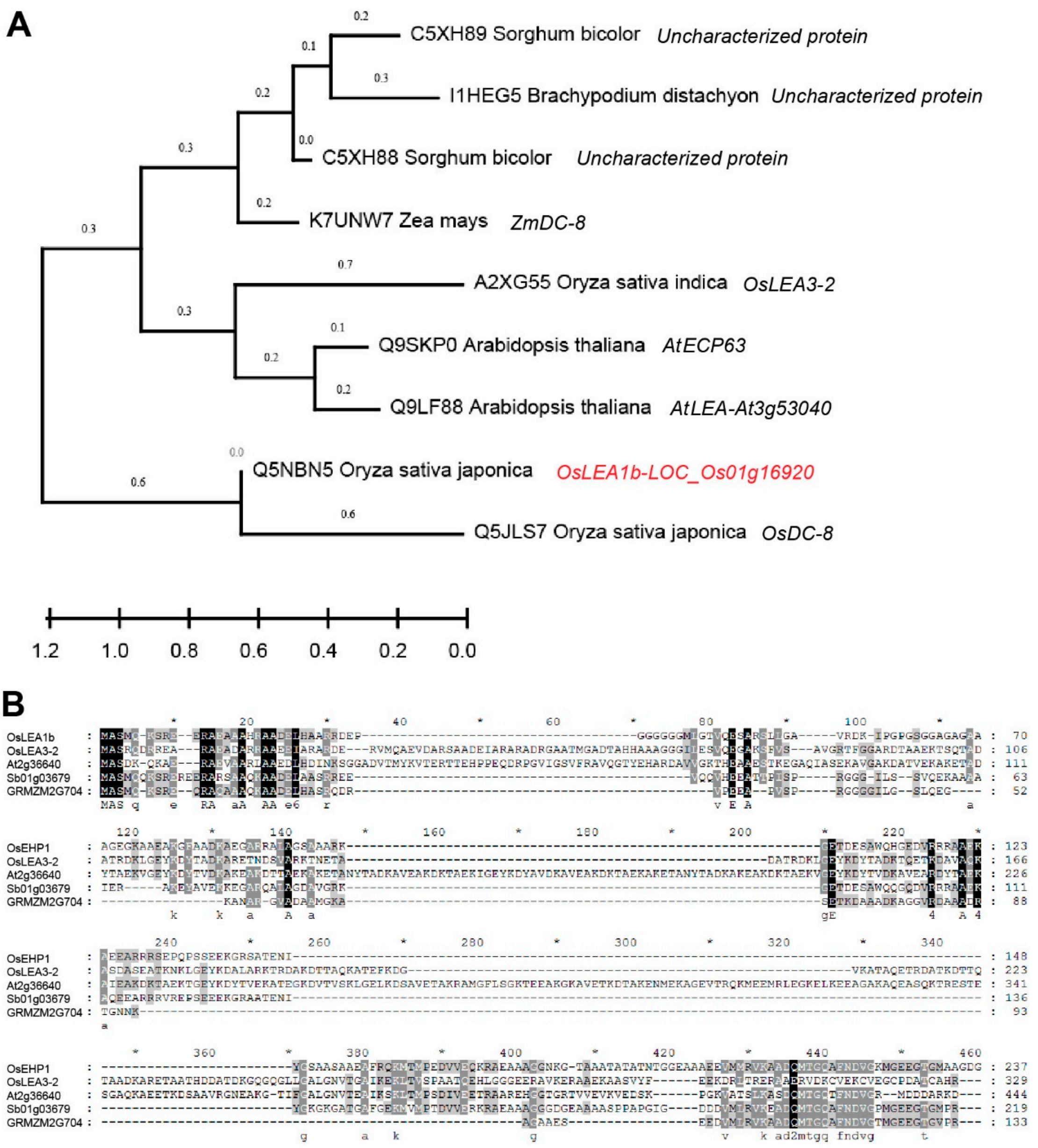

Figure S1. Phylogenetic analysis and amino acid sequence alignment of OsLEA1b.

(A) Phylogenetic analysis of *OsLEA1b*. The neighbor-joining tree was generated with MEGA7 after aligning the full-length protein sequences. Distances are evaluated using the neighbor-joining algorithm. **(B)** Amino acid sequence alignment between part of OsLEA1b and other homologous proteins. The sequences alignment was generated with ClustalX2. White alphabet with black background means 100% identity, white alphabet with grey background represents 80% identity, and black alphabet with light grey background stands for 60% identity.

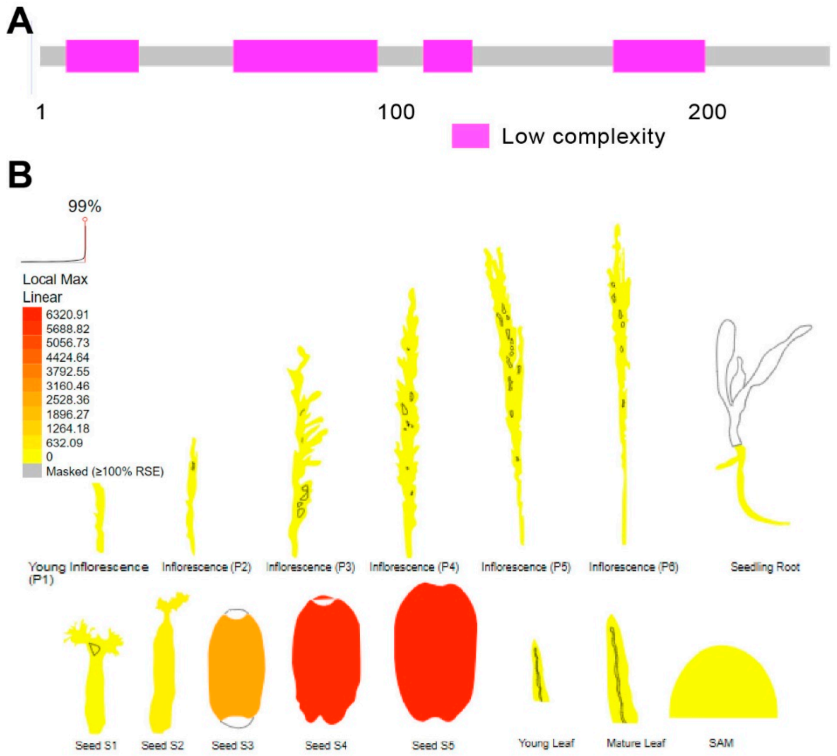

Figure S2. Structure and expression pattern prediction of *OsLEA1b*.

(A) Predicted structure of OsLEA1b from website (http://smart.embl-heidelberg.de). Pink box represent the low complexity region. **(B)** Tissue expression pattern prediction of *OsLEA1b* on the website (https://bar.utoronto.ca/eplant_rice/).

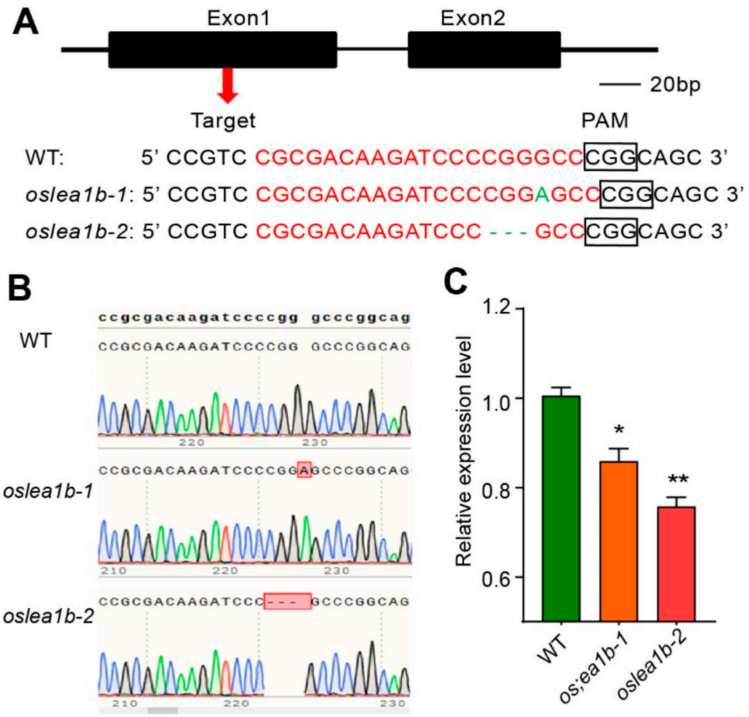

Figure S3. CRISPR/Cas9 mediated editing lines of *OsLEA1b*.

(A, B) Gene structure of *OsLEA1b* and sequencing of CRISPR/Cas9 target region in WT and T_0 plants. Scale bars, 20 bp. **(C)** Relative expression level of *OsLEA1b* in transgenic knock out plants (*oslea1b-1,2*). Data are means ± SD from not less than three biological replicates. Statistically significant differences were determined using Student's *t*-test (*, $P < 0.05$, **, $P < 0.01$).

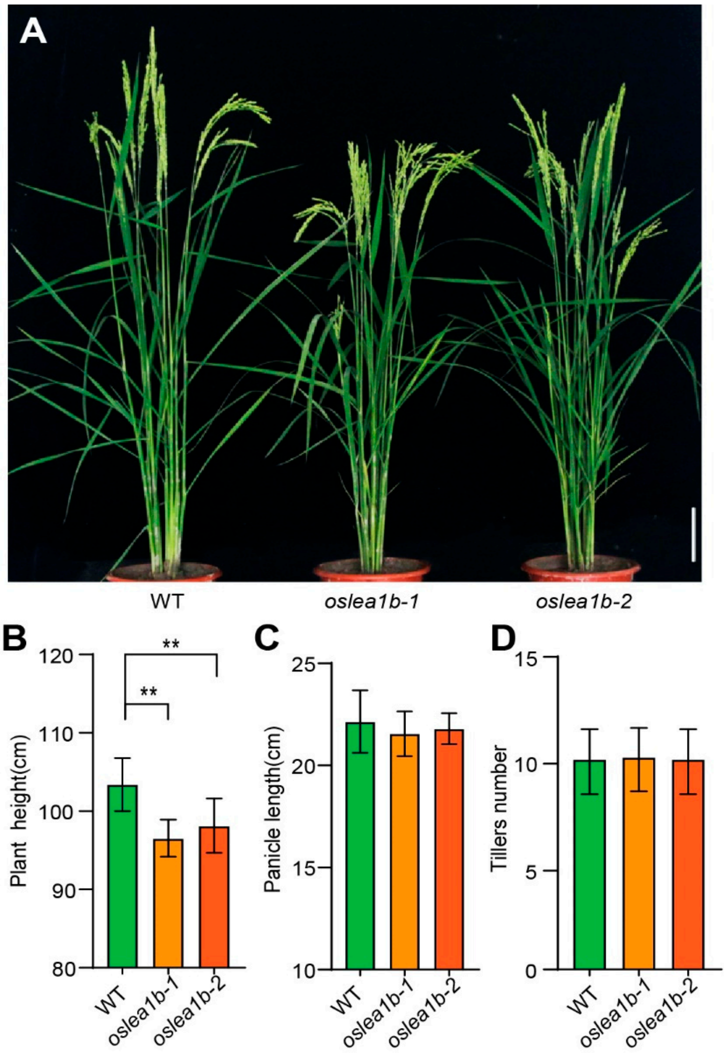

Figure S4. Appearance analysis of WT and *oslea1b* mutants.

(A) The plant phenotype of WT and *oslea1b* mutants after heading. Scale bars, 15 cm. **(B-D)**. Statistics of plant height, panicle length and tillering number of wild type and mutants. Data are means ± SD from not less than three biological replicates. Statistically significant differences were determined using Student's *t*-test (**, $P < 0.01$).

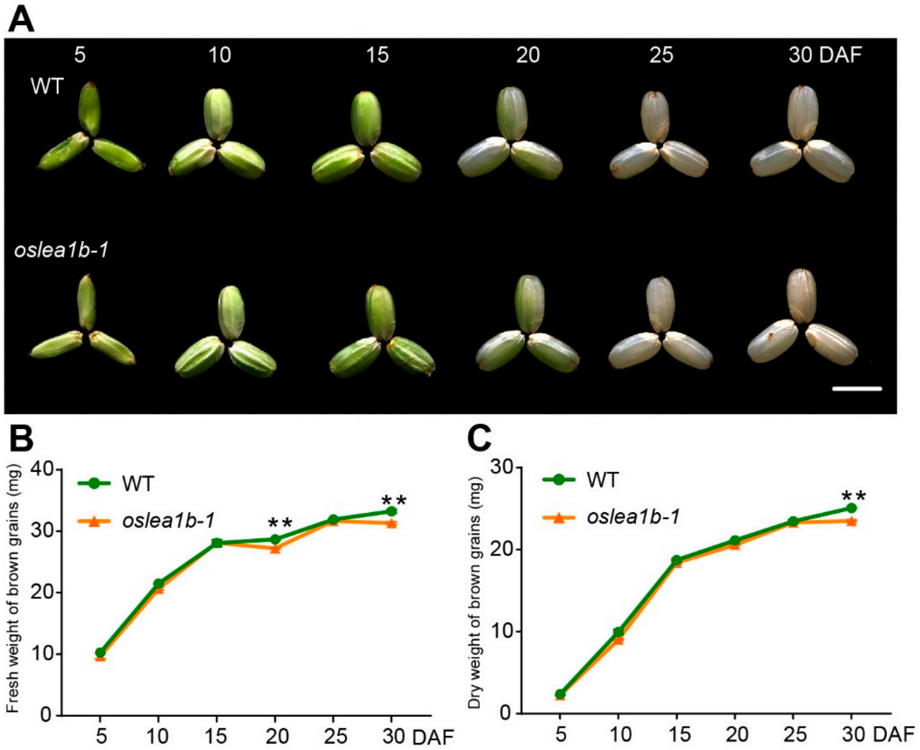

Figure S5. Grains filling of WT and *oslea1b* mutants.

(A) Fresh grains of WT and *oslea1b* at various stages of development. DAF, days after fertilization. Scale bars, 5 mm. **(B, C)** Weight of fresh and dry grains of WT and *oslea1b* at various stages of grain filling. Data are means ± SD from not less than three biological replicates. Statistically significant differences were determined using Student's *t*-test (**, $P < 0.01$).

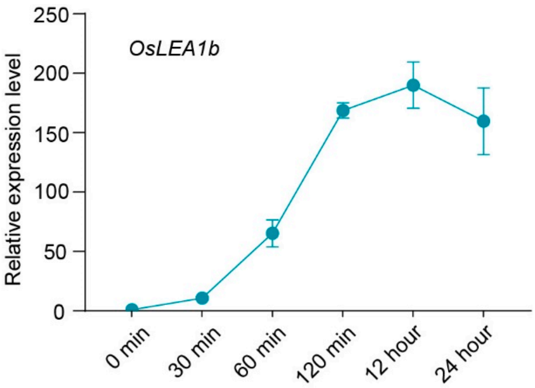

Figure S6. High temperature induces the expression of *OsLEA1b*.
Relative expression level of *OsLEA1b* after high temperature treatment within 24 hours.

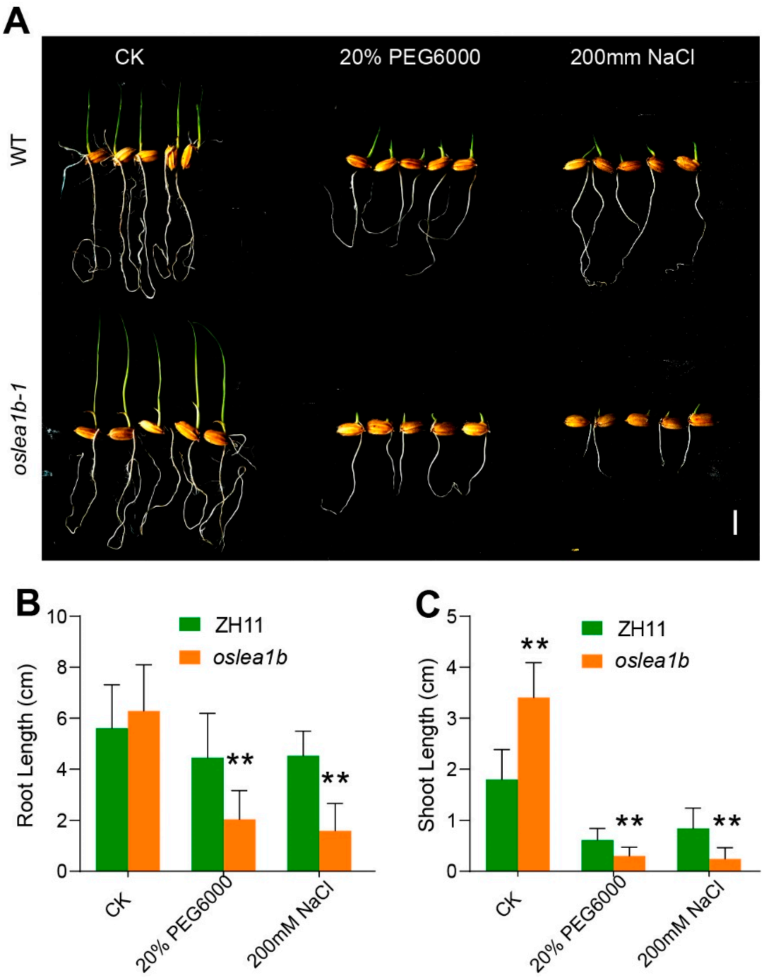

Figure S7. OsLEA1b is a positive regulator of drought tolerance.

(A) The phenotype of WT and *oslea1b* mutants under 20% PEG6000 and 200 mM NaCl treatment for 1 week. Scale bars, 5 mm. **(B, C)** The root and shoot length of the *oslea1b* mutants under 20% PEG6000 and 200 mM NaCl treatment for 1 week. Data are means ± SD from not less than three biological replicates. Statistically significant differences were determined using Student's *t*-test (**, $P < 0.01$).

Table S1. Primers used in this study

Use	Primer name	Sequence (5' to 3')
CRISPR/Cas9	Cas9-OsLEA1b-F	CAGCGCGACAAGATCCCCGGGCC
	Cas9-OsLEA1b-R	AACGGCCCGGGGATCTTGTCGCG
	Cas9-OsLEA1b-check-F	AAGCAGAGAGCGAAGCAAGCTCGAA
	Cas9-OsLEA1b-check-R	GCTACTTCTCCTCCGACGACGGCTGAG
GUS expression	1305-GUS-OsLEA1b-F	CCATGATTACGAATTCTATCCTTACAAGTTTGTTT
	1305-GUS-OsLEA1b-R	CTCAGATCTACCATGGTGATCAGATGATCTTGGTC
Subcellular localization	OsLEA1b-GFP-F	GGTACCTGCAACTAGTATGGCGTCCATGCAGAAGA
	OsLEA1b-GFP-R	TGCTCACCATGGATCCGCGGCGGCGCCCACCATCT
q-RT-PCR	OsLEA1b-F	CGGCGACGGAGAACATCTA
	OsLEA1b-R	CTCCGGCATCGTCATCTTCT
	BEI-F	TGGCCATGGAAGAGTTGGC
	BEI-R	CAGAAGCAACTGCTCCACC
	SSIIIa-F	GCCTGCCCTGGACTACATTG
	SSIIIa-R	GCAAACATATGTACACGGTTCTGG
	PUL-F	ACCTTTCTTCCATGCTGG
	PUL-R	CAAAGGTCTGAAAGATGGG
	ISA3-F	ACAGCTTGAGACACTGGGTTGAG
	ISA3-R	ACAGCTTGAGACACTGGGTTGAG
	GBSSI-F	TCCGTCATTCCTGGAGAA
	GBSSI-R	CTTTGAAGTATGGGTTGTTGT
	BEIIb-F	ATGCTAGAGTTTGACCGC
	BEIIb-R	AGTGTGATGGATCCTGCC
	OsBT1-F	GAGGTCTTCCGCTGGATCAT
	OsBT1-R	TGAAATGCTCGATGGCCTTG
	SBE1-F	GCTGAAGATGTTTCGGGCAT
	SBE1-R	CATTGACCATTTGCGGTCCT
	RAG2-F	TGCTCCTCATCATCGTCTCC
	RAG2-R	TGCTCCTCATCATCGTCTCC
	GLUD1-F	TGAACCAAGGCAGAAGGAGT
	GLUD1-R	GCCTCGCTAAGTACCTCAGT
